# Tomato Metabolic Changes in Response to Tomato-Potato Psyllid (*Bactericera cockerelli*) and Its Vectored Pathogen *Candidatus* Liberibacter solanacearum

**DOI:** 10.3390/plants9091154

**Published:** 2020-09-06

**Authors:** Jisun H.J. Lee, Henry O. Awika, Guddadarangavvanahally K. Jayaprakasha, Carlos A. Avila, Kevin M. Crosby, Bhimanagouda S. Patil

**Affiliations:** 1 Vegetable and Fruit Improvement Center, Texas A&M University, 1500 Research Parkway, A120, College Station, TX 77845-2119, USA; jslee@tamu.edu (J.H.L.); gkjp@tamu.edu (G.K.J.); 2 Department of Horticultural Sciences, Texas A&M University, College Station, TX 77843, USA; 3 Texas A&M AgriLife Research and Extension Center, 2415 E Hwy 83, Weslaco, TX 78596, USA; Henry.Awika@ag.tamu.edu

**Keywords:** tomato, *Bactericera cockerelli*, *Candidatus* Liberibacter solanacearum, UPLC/HR-QTOF-MS, HS-SPME/GC-MS, phenolics, hormones

## Abstract

The bacterial pathogen ‘*Candidatus* Liberibacter solanacearum’ (Lso) is transmitted by the tomato potato psyllid (TPP), *Bactericera cockerelli*, to solanaceous crops. In the present study, the changes in metabolic profiles of insect-susceptible (cv CastleMart) and resistant (RIL LA3952) tomato plants in response to TPP vectoring Lso or not, were examined after 48 h post infestation. Non-volatile and volatile metabolites were identified and quantified using headspace solid-phase microextraction equipped with a gas chromatograph-mass spectrometry (HS-SPME/GC-MS) and ultra-high pressure liquid chromatography coupled to electrospray quadrupole time-of-flight mass spectrometry (UPLC/ESI-HR-QTOFMS), respectively. Partial least squares-discriminant analysis (PLS-DA) was used to define the major uncorrelated metabolite components assuming the treatments as the correlated predictors. Metabolic changes in various classes of metabolites, including volatiles, hormones, and phenolics, were observed in resistant and susceptible plants in response to the insects carrying the pathogen or not. The results suggest the involvement of differentially regulated and, in some cases, implicates antagonistic metabolites in plant defensive signaling. Upon validation, the identified metabolites could be used as markers to screen and select breeding lines with enhanced resistance to reduce economic losses due to the TPP-Lso vector-pathogen complex in Solanaceous crops.

## 1. Introduction

The phloem-limited bacterium *‘Candidatus* Liberibacter solanacearum’ (Lso) is vectored by the tomato-potato psyllid (TPP, *Bactericera cockerelli*), causing severe symptoms on Solanaceae crops, including Zebra Chip disease in potato (*Solanum tuberosum*) and vein greening in tomato (*Solanum lycopersicum*) [1,2,3]. Some wild tomato relatives as germplasm resources have been screened for polygenic resistance traits against the TPP [4]. Particularly, several major quantitative trait loci (QTLs) related to adult TPP mortality and fecundity were confirmed in *Solanum habrochaites* using recombinant inbred lines (RILs) [5]. Major resistance QTLs found in the RIL LA3952 carrying the *S. habrochaites* insertion on chromosome 8 revealed that the presence of Lso is associated with increased adult TPP mortality, while the reduced TPP oviposition trait in LA3952 is independent of Lso [5].

Thus far, substantial genomic analysis has underscored the influence of the TPP-Lso complex in potato and tomato genotypes [6,7,8,9], and constitutive and elicited phytohormones, such as jasmonic acid and salicylic acid have been reported to play crucial roles in the host plants by deploying defensive signaling pathways involved in the host resistance against insect herbivores and pathogens [6,7,8,9]. For instance, volatile metabolites, such as monoterpenes play a role in response to insect herbivory by attracting carnivorous predators as an indirect plant defensive mechanism [10,11,12]. Conversely, the phenolic p-coumaric acid inhibits the type III secretion system (T3SS) virulence genes of the gram-negative plant pathogen *Dickeya dadantii* [13]. For phytohormones, their regulation varies, and may be specific to host and pathogenic strains [14]. To fully understand this may require that the response to stress by the vast majority of metabolites and phytochemicals be delineated and if possible, be predictable enough to enable the long-term application in resistance breeding programs. Little information is known about responses of susceptible and resistant tomato genotypes to the TPP carrying Lso pathogen [9,15,16]. Metabolomics approaches using liquid chromatography (LC) and gas chromatography (GC) coupled with mass spectrometry (MS) are some of the effective present-day tools for identifying and quantifying plant metabolites, including phenolics, phytohormones, volatile organic compounds (VOCs), in the induced response to pathogens and screening potential biomarkers [17,18,19].

Herbivore-induced changes of metabolite profiles could be influenced by several factors, including developmental the presence of the pathogen, stages of plants, insect species, infestation pressure, and different periods after inoculation [20,21,22,23]. The added intervention of the pathogen has created a complex interaction between host, insect, and pathogen [24], which is worth multifaceted studies in order to improve our understanding of the plant’s innate immune responses to vector colonization and pathogen infection [9,16,19]. In the present study, we report a comparative tomato metabolic profile in response to TPP carrying the Lso pathogen or not in resistant and susceptible tomato genotypes in order to identify putative metabolites involved in defensive signaling in the TPP-Lso-tomato complex interaction.

## 2. Results

### 2.1. Untargeted Metabolomic Analysis Showcases the Global Differential Induction of Non-Volatile Metabolites in the Presence of TPP or TPP with Lso

**Figure 1:**
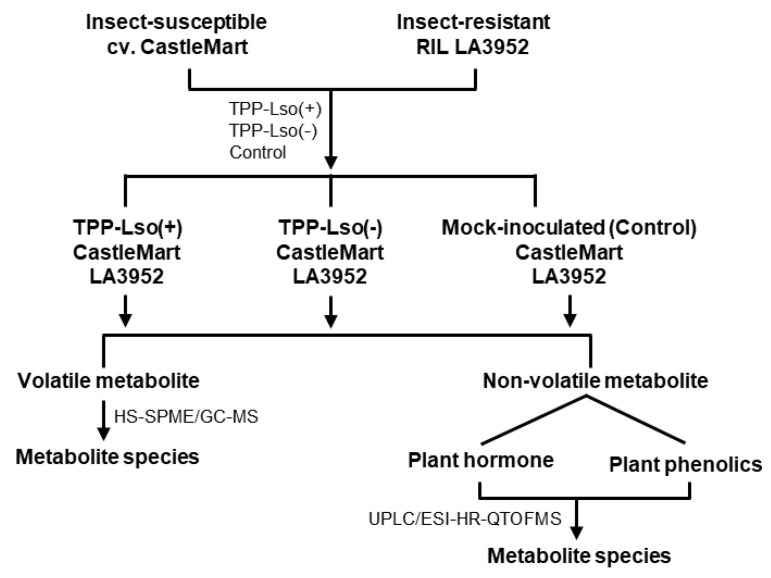
Experimental design for the investigation. The insect-susceptible (CastleMart) and insect-resistant resistant (RIL LA3952) tomato plants were inoculated with tomato potato psyllid (TPP) carrying or not the *Candidatus* Liberibacter Solanacearum (Lso). The five-week-old tomato seedlings were infested and harvested two days after infestation. Five replications were used for each treatment.

**Figure 2:**
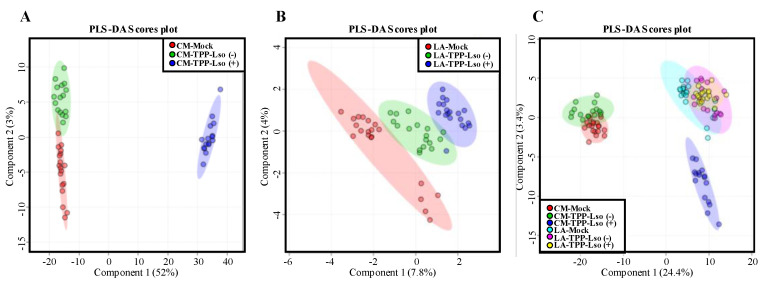
Two-dimensional partial least squares discriminate analysis (PLS-DA) of untargeted metabolomics. The dataset was obtained by ultra-performance liquid chromatography coupled to electrospray ionization high resolution quadrupole time-of flight mass spectrometry (UPLC/ESI-HR-QTOFMS). The PLS-DA score plots depict the differential metabolite clustering according to the treatments CM_TPP-Lso(−), CM_TPP-Lso(+) and CM_Mock as a control for insect-susceptible tomato genotype CastleMart,CM (**A**) and the treatments LA_TPP-Lso(−), LA_TPP-Lso(+), and LA_Mock as control for insect-resistant RIL LA3952, LA (**B**). A combined test for both genotypes and test groups is shown in (**C**).

In this study, the change of volatile and non-volatile metabolites of insect-susceptible (cv CastleMart) and resistant (RIL LA3952) tomato plants in response to Lso-positive or Lso-negative adult psyllids was assessed after 48 h post infestation (Figure 1). Initially, we determined the global metabolic abundance between test groups by an untargeted metabolomic dataset derived from UPLC/ESI-HR-QTOFMS. Partial least squares-discriminant analysis (PLS-DA) score plots were used to showcase the metabolite profiles in two tomato genotypes, the susceptible CastleMart (CM) and a resistant LA3952 (LA) inoculated with Lso-free and Lso-positive TTP, [TPP-Lso(−) and TPP-Lso(+), respectively] (Figure 2). With reference to the mock (Control) plants, there were distinct metabolic profile clusters for the CM_TPP-Lso(−) different from CM_TPP-Lso(+) (Figure 2A). Similarly, metabolite profiles of LA_TPP-Lso(−) were separated from those of LA_TPP-Lso(+) and control groups (Figure 2B). The results imply that some insect-associated metabolites may be controlled differentially from those induced metabolites in the presence of the pathogenic bacteria (Lso+) on the insect in each of the two tomato genotypes. To evaluate the assumption of genotypic non-correlation between CM and LA3952, we determined the PLS components in a multivariate analysis combining metabolic profiles for all predictors (treatments) for the CM and LA3952 plants (Figure 2C). The responses to TPP and TPP-Lso were regrouped into three distinct classes, and the first cluster consists of metabolites associated with susceptible CastleMart, CM_TPP-Lso(−) being closely clustered with a subgroup of the CM_Mock control. The second main cluster consisted of metabolites of resistant LA3952 plants in response to the mock control, TPP-Lso(−), and TPP-Lso(+) inoculation. The third distinct cluster was metabolites of the CM_TPP-Lso(+). These observations suggest that inoculation-linked metabolite alterations could be influenced by the presence of the pathogen Lso in the insect which may elicit some metabolite species distinctively. Interestingly, the insect TPP alone or the insect with Lso can induce more similar metabolite species in the resistant LA.

### 2.2. Phenolic Metabolites in Tomato Show Both Constitutive Genotype-Dependent and Treatment-Specific Induced Differences in Response to TPP and Lso

**Figure 3:**
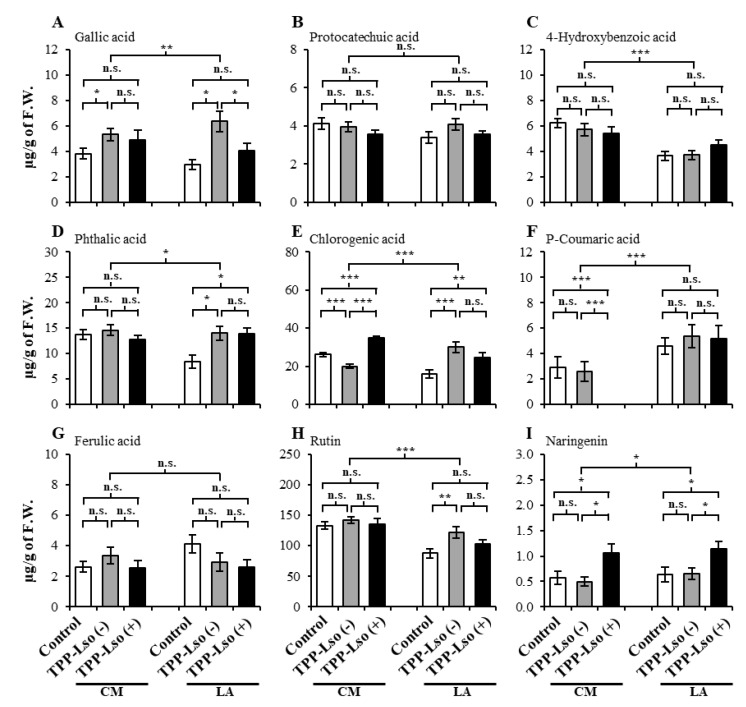
The levels of phenolics in the susceptible CastleMart (CM) and resistant RIL LA3952 (LA) tomato genotypes according to treatments, mock control, TPP-Lso(−), and TPP-Lso(+). (**A**) gallic acid, (**B**) protocatechuic acid, (**C**) 4-hydroxybenzoic acid, (**D**) phthalic acid, (**E**) chlorogenic acid, (**F**) p-coumaric acid, (**G**) ferulic acid, (**H**) rutin, and (**I**) naringenin. The results are presented as mean ± S.E., *, **, and *** denote significant difference at *p* ≤ 0.05, ≤ 0.01, and ≤ 0.001, respectively; ns, not significant. The means were separation by the Tukey’s honestly significant difference (HSD) test.

Nine phenolic compounds, including gallic acid, protocatechuic acid, 4-hydroxybenzoic acid, phthalic acid, chlorogenic acid, p-coumaric acid, ferulic acid, rutin, and naringenin were identified and quantified using UPLC/ESI-HR-QTOFMS (Table S1). The phenolic levels per treatment are presented in Figure 3. The expressed abundance of some of the nine phenolic compounds were genotype-dependent as well as treatment-specific in a manner that suggests that the upregulation of these compounds is influenced by the insect and the interaction with the bacteria, that there exists basal resistance in the susceptible CM, that genotype-specific constitutive resistance factors may be ubiquitous in tomato plants. Finally, different phenolics widely associated with plant defense may have antagonistic roles in susceptible versus resistant plant genotypes.

For instance, both 4-hydroxybenzoic acid, p-coumaric acid, and rutin (Figure 3C,F,H, respectively) mainly were genotypically influenced. The abundances of 4-hydroxybenzoic acid and rutin were not significantly different under all treatments in the susceptible CM, but were significantly lower in the resistant LA3952 under all treatments, suggesting that 4-hydroxybenzoic acid and rutin may be playing antagonistic roles in the resistance against the insect in the presence or absence of the bacterial pathogen, and can be selected against in developing resistance. Similar but opposite accumulation patterns were observed for the p-coumaric acid (Figure 3F), where the constitutive abundance in CM was significantly lower than the constitutive abundance in LA3952 while within-genotype differences remained insignificant, suggesting that p-coumaric acid may be important in improving resistance. More dramatically, p-coumaric acid was not detected in the CM challenged with TPP_Lso(+), suggesting that its production was severely suppressed specifically in the presence of the pathogen Lso in the susceptible CM.

### 2.3. Defense Hormones and Signaling Molecules Show Constitutive Abundance Determined by the Presence of TPP and/or Lso in Susceptible and Resistant Tomato

**Figure 4:**
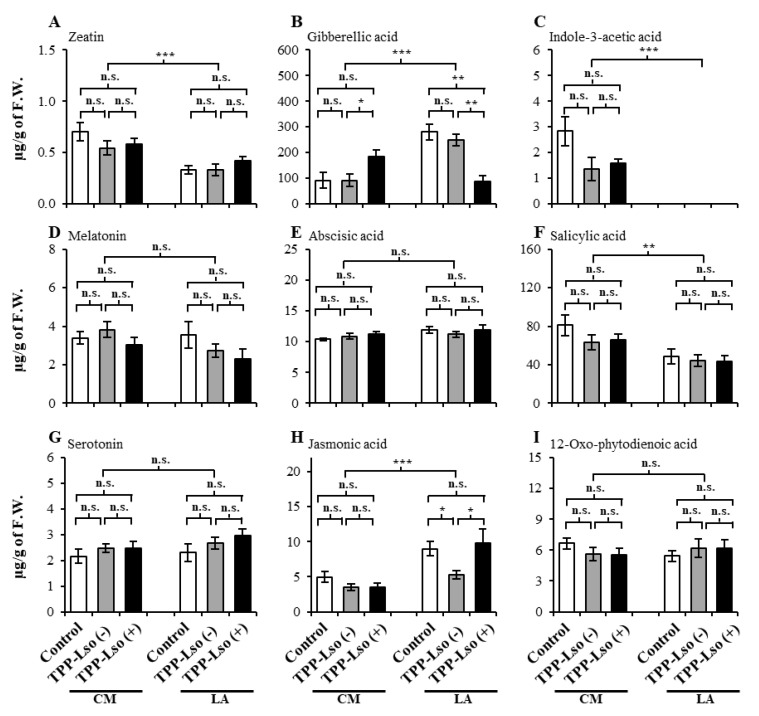
The levels of hormones and other signaling molecules in insect-susceptible CastleMart (CM) and -resistant RIL LA3952 (LA). (**A**) zeatin, (**B**) gibberellic acid, (**C**) indole-3-acetic acid, (**D**) melatonin, (**E**) abscisic acid, (**F**) salicylic acid, (**G**) serotonin, (**H**) jasmonic acid, and (**I**) 12-oxo-phytodienoic acid. The results are expressed as mean ± S.E. *, **, and *** denote significant at *p* ≤ 0.05, ≤ 0.01, and ≤ 0.001, respectively; ns, not significant.

A total of six plant hormones zeatin, gibberellic acid, indole acetic acid, abscisic acid, salicylic acid, jasmonic acid, and other three plant signaling compounds, 12-oxophytodienoic acid (OPDA), melatonin, and serotonin from tomato plants were analyzed using UPLC/ESI-HR-QTOFMS (Table S2). Just as observed with the phenolics, the abundance of the hormone, abscisic acid (ABA, Figure 4E), and signaling molecules melatonin (Figure 4D), serotonin (Figure 4G), and 12-oxo-phytodienoic acid (Figure 4I) showed detectable but not statistically different abundance between genotypes or treatments, suggesting their constitutive levels are not influenced by the TPP in the presence or absence of Lso in the 48 h after inoculation.

Others such as zeatin (Figure 4A), a cytokinin known to encourage lateral growth of buds and salicylic acid—SA (Figure 4F), a well-documented plant defense signaling hormone, surprisingly, were constitutively high in the susceptible CM but constitutively low in the resistant LA3952 under all treatments, suggesting that these compounds might not have played a key role in the defense against the TPP-Lso complex at 48 h after inoculation. The dramatic absence of indole-3-acetic acid, IAA, in the resistant LA3952 (Figure 4C) with no observable breach in growth was, particularly puzzling since IAA is a well-reported auxin, which is important in plant growth functions.

Other plant hormones such as gibberellic acid, GA (Figure 4B) and jasmonic acid, JA (Figure 4H) showed constitutively higher levels in the resistant LA3952 than in the susceptible CM, suggesting a putative role in defensive signaling. However, the presence of Lso in TPP may inversely regulate the accumulation of these two plant hormones. For example, GA abundance in the LA_TPP-Lso(+) plants were significantly lower than those of the LA_Control, LA_Lso(−) and in CM_TPP-Lso(+). Conversely, JA levels were substantially lower in LA_TPP-Lso(−) than in the LA_control and in LA_Lso(+). Together, the data suggest that the JA is slightly suppressed by psyllid activity in the absence of Lso, while GA may be suppressed in the presence of Lso in the resistant LA genotype. Therefore, the presence or absence of psyllids and Lso may indirectly regulate defensive signaling against TPP by influencing plant hormone accumulation. Likewise, it has been previously reported that TPP mortality in resistant LA plants is reduced in LA_TPP-Lso(−) infested plants [5]. However, further analysis needs to be performed to determine if the observed changes in JA and GA changes are associated to lower mortality in the LA resistance plants.

### 2.4. Only a Handful of Volatile Metabolites of the Fatty Acid Derivatives, Monoterpene, Norisoprenoids, Phenylpropanoid-Derivatives and Sesquiterpenes Show Significantly Altered Abundance Associated with Resistance to TPP

**Figure 5:**
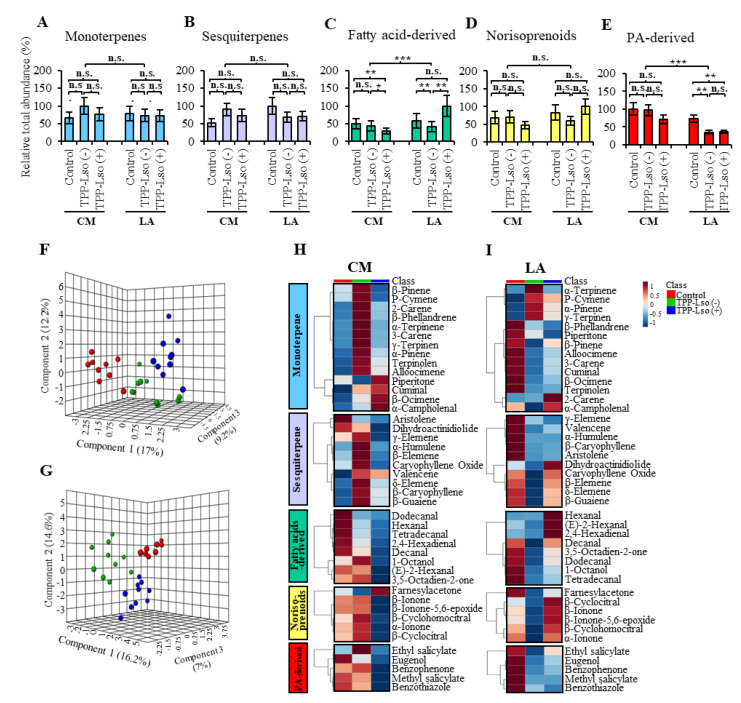
Volatile metabolite abundance in Control, TPP-Lso(−), and TPP-Lso(+) infested tomato plants, CastleMart (CM), and RIL LA3952 (LA), respectively. (**A**) monoterpenes (**B**) sesquiterpenes (**C**) fatty acid-derived (**D**) norisoterpenoid (**E**) phenylpropanoids (PA)-derived. Bar graphs indicate the mean total abundance of detected compounds in each chemical class. Three-dimensional (3D) sparse partial least square-discriminant analysis (sPLS-DA) scores plots of (**F**) insect-susceptible (CM) and (**G**) insect-resistant (LA) tomato plants demonstrate that each tomato variety can be separated into distinct groups without overlap according to the treatment [Blue dots, TPP-Lso(+); green, TPP-Lso(−), and red, mock-infested control]. The heatmaps show the effect of treatment on the mean abundance of studied metabolites within groups based on tomato varieties such as CM (**H**) and LA (**I**). The scale legend of the heatmap indicates the relative abundance of volatile metabolites in each chemical group. *, **, and *** denote significant at *p* ≤ 0.05, ≤ 0.01, and ≤ 0.001, respectively; n.s., not significant.

A total of 43 volatile metabolites (Table 1) were identified and grouped into five main chemical classes namely monoterpene, sesquiterpene, fatty acids-derived, norisoprenoids, and phenylpropanoids (Table 1, Figure 5A–E). At the class level, there were only two groups, fatty acid (FA)-derived and phenylpropanoid (PA)-derived, which showed significant within-class mean differences between genotypes and between treatments. The FA-derived group showed the smallest mean values in the CM_TPP-Lso(+) compared to the CM_Control and CM_TPP-Lso(−), while in the resistant LA3952, the FA-derived group had the largest mean abundance in the LA_TPP-LSo(+) among the treatments (Figure 5C), suggesting that the genes regulating some species of VOCs derived from FA are disproportionately highly induced in the presence of the pathogen Lso. On the other hand, the mean levels of phenylpropanoid (PA)-derived species were significantly larger in both the LA_TPP-Lso(−) and LA_TPP-Lso(+) plants than in the LA_Control; levels in the LA_Control showed no statistical difference compared to those of all the treatments in CM plants, suggesting that some of the PA-derived species of VOCs are disproportionately down-regulated in the presence of the TPP feeding.

In fact, among the eight FA-derived VOC species, the mean differences noted above were determined mainly by (E)-2-hexenal and hexanal. (E)-2-hexenal was the most abundant, between a low of 230.63 ± 22.94 (CM-Control), 223.99 ± 42.87 in CM_TPP-Lso(−), 141.97 ± 17.01 in CM_TPP-Lso(+), and in the resistant LA3952 at 492.11 ± 51.39 (LA_TPP-Lso(+) compared to the 309.03 ± 43.91 (~40% reduction) in LA_TPP-Lso(−) and the 257.62 ± 31.03 (~48% lower) in the LA-Control. In the same group, hexanal was the only other FA-derived metabolite that showed a similar trend though at a much-reduced abundance (Table 1). The rest six FA-derived VOCs showed lower abundances (<3.1) with no significant differences between the genotypes or between treatments. Similar trends, although at much-reduced abundance were also observed in the norisoprenoid, β-Ionone while the rest of the species in this group did not show any significant differences between the test groups, suggesting that the FA-derived (H)-2-hexenal and hexanal and the norisoprenoid, β-Ionone, maybe the three main volatile metabolites in this study that were associated with resistance against the pathogen Lso.

For the mean PA-derived group showing down-regulation in resistant LA_TPP-Lso(−) and LA_TPP-Lso(+), four out of five compounds had their abundances significantly lower in the two treatments compared to all the other treatments in CM and LA_Control.

Methyl-salicylate showed the most notable abundance which was at a high of 10.17 ± 1.52 (CM_Control), 9.38 ± 1.08 CM_TPP-Lso(−), 7.22 ± 1.14 CM_TPP-Lso(+) and 5.99 ± 0.63 for LA_Control and a significantly reduced abundance of 2.78 ± 0.47 in LA_TPP-Lso(−) and 2.76 ± 0.22 in LA_TPP-Lso(+). Other compounds, namely eugenol, benzophenone, and benzothiazole, showed a very similar trend, albeit at significantly lower corresponding abundances, suggesting that these compounds may be influenced by susceptibility factors induced during TTP feeding or Lso interaction in the susceptible CM plants.

Among the 14 monoterpenes, only β-phellandrene showed significant difference in abundance, which was between CM_TPP-Lso(−) (701.74 ± 75.28) and all the other treatment sets: 476.35 ± 40.23 in CM_Control, 519.46 ± 74.65 in CM_TPP-Lso(+), 538.26 ± 51.54 LA_Control, 503.77 ± 39.98 in LA_TPP-Lso(−), and 493.63±47.56 in LA_TPP-Lso(+). This suggests that β-Phellandrene is not only ubiquitously abundant but may be a marker for insect susceptibility in the CM. Similar trends though at much-reduced and insignificant differences in abundance were observed in the sesquiterpene, β-Caryophyllene. Displays of all the 43 volatile metabolites investigated in this study are depicted in bar graphs, sparse partial least squares discriminant analysis (sPLS-DA) score plots, and heat maps, according to the treatment to showcase the abundance of five volatile classes (Figure 5). The sPLS-DA models identified component 1, 2, and 3 to differentiate between treatment groups, and Figure 5F,G indicate that each treatment in both tomato plants has distinct separation, accounting for 38.4% and 37.8% of the total variation, respectively.

## 3. Discussion

The main objective of the present study was to gain insight into the metabolite profiles of insect-susceptible and resistant tomato plants in response to the infestation with tomato-potato psyllids (TPP), carrying or not *Candidatus* Liberibacter solanacearum (Lso).

### 3.1. Tomato Phenolic Composition Is Differentially Regulated in Susceptible and Resistant Plants in Response to TPP-Lso Infestation

The phenolic pathways, including those involving the nine compounds in the present study, have been previously implicated in the plant defensive mechanism against TPP carrying the Lso in Solanaceae crops [16,20]. However, most of the phenolic compounds in our study did not profile differentially enough to unambiguously distinguish the expected resistance responses between the LA3952 and the insect susceptible CastleMart (CM). On this basis, the compounds could broadly be categorized into three. In the first category are those that did not show significant differences between treatments [TPP-Lso(−), TPP-Lso(+) and the Mock as control], and between the two genotypes (insect-susceptible CM and resistant LA3952). Compounds such as protocatechuic acid, phthalic acid, and ferulic acid (Figure 3B,C,G, respectively) were not significantly different between the two genotypes (CM and LA3952) or between the TPP-Lso treatments, although they have previously been implicated in defensive signaling in other plant-psyllid interaction systems. For example, a study on the effects of the carrot psyllid, *Trioza apicalis*, on phenolics reported that the change in ferulic acid levels could be affected by experimental conditions, such as insect density and plant growth stage [21]. They did not detect the presence of any phytoplasma associated with Lso in laboratory psyllid colonies or in the carrot seedling leaves. However, one of their earlier studies had established an association between *T. apicalis* and Lso from both field and laboratory raised samples [25].

The second category includes compounds that showed significant treatment effects, and thus suggests the influence of TPP feeding and Lso on the phenolic profiles, but without significant differences that we expected between LA3952 and the susceptible CM. For instance, gallic acid, chlorogenic acid, rutin, and naringenin Figure 3A,E,H,I, respectively) fell in this category. Gallic acid, which showed increased abundance in the insect-inoculated plants of both genotypes, has previously been reported to be involved in the plant defense system, due to its toxicity to the development and growth of melon fruit fly (*Bactrocera cucurbitae*) [26]. Similarly, chlorogenic acid has been shown to inhibit the growth of a wide spectrum of bacteria [27] and elevated levels in leaves of *Solanum lycopersicum* offer resistance against the bacterial pathogen *Pseudomonas syringae* pathovar Tomato [28] and against *Spodoptera litura* has previously pointed to its anti-herbivore activity [29]. In our experiment, chlorogenic acid showed significantly elevated levels in the CM_TPP-Lso(+) and inoculated LA treatments regardless of insect resistance or susceptibility. In fact, compounds like naringenin (Figure 3I) showed very significantly elevated levels in the TPP-Lso(−) plants compared to the control plants in both the LA3952 and CM, suggesting their involvement in induced basal resistance response. The third category shows clear genotypic differences in the phenolic profiles that suggest either treatment-induced or constitutive resistance difference between LA3952, and the susceptible CM. Variation of metabolite levels with respect to genotype and treatment were observed among studied nine phenolic metabolites, and p-coumaric acid may confer the resistance in RIL LA3952 by associated plant defense mechanism [30,31,32].

In this group, only two compounds, 4-hydroxybenzoic acid (Figure 3C), and p-coumaric acid (Figure 3F), seem to significantly demarcate the difference between the resistance of the two genotypes. It has been previously reported that p-coumaric acid may contribute to plant resistance in response to the pathogenic fungus Fusarium [33], and the facultative anaerobe plant pathogenic bacterium *Dickeya dadantii* [13]. Indeed, we observed dramatically higher levels of p-coumaric acid in the resistant LA3952 plants compared to susceptible plants. In addition, the undetectable level of this metabolite in the CM_TPP-Lso(+) plants (Figure 3F) suggests that it may be comparatively severely down-regulated in response to Lso (+) in susceptible plants, leading us to speculate that p-coumaric acid is constitutively enhanced in resistant plants, and may be a good metabolite biomarker for resistance against both the insect TPP and its transmitted pathogen, the Lso. Instructively, the dissimilar but constitutively significant elevation of 4-hydroxybenzoic acid (lower abundance in the resistant) and p-coumaric acid suggest that the genes regulating these two compounds may be antagonistic, with 4-hydroxybenzoic acid representing susceptibility factors and p-coumaric acid, representing the constitutive presence of resistance factors.

Additionally, since naringenin (Figure 3I) was significantly elevated only in the TPP-Lso(+)-inoculated CM and LA3952, while the levels were similarly low in both the TPP-Lso(−) and the Control tests for both genotypes, we suggest that naringenin is associated with induced resistance specific to TPP insects carrying the pathogen bacteria Lso in both genotypes and in conjunction with the p-coumaric acid, may be important in identifying and disambiguation of induced resistance to the pathogen Lso.

### 3.2. Differential Constitutive, and in Some Cases Antagonistic Alternating Roles of Plant Hormones May be Implicated in the Unexpected Contrasting Abundances

Certain hormones such as cytokinin, gibberellic acid, auxin, and abscisic acid have been known to play important regulatory roles in plant development, growth [34], and plant immune responses [35]. We determined that the differential abundance of some of these plant hormones may be indicative of their contrasting responses and roles in plants infested with the psyllid *Bactericera cockerelli* and psyllids carrying the pathogenic bacterium *Candidatus* liberibacter solanacearum (Lso). Gibberellic acid (GA), for instance, has been reported to have a negative role in rice basal disease resistance to the bacterial blight disease [36], and we speculate this might explain the down-regulation of GA in the LA_TPP-Lso(+) as opposed to the LA_Control and the LA_TPP-Lso(−). In fact, there has been evidence to suggest that some bacterial and fungal pathogens produce GA, which may, in some cases, implicate this metabolite in avirulent activity [36]. In contrast, the GA levels were significantly up-regulated in the susceptible CM plants infested with TPP carrying Lso compared to the control and TPP-Lso (−) plants, suggesting that in this experiment, GA’s role in resistance may be constitutively insect-specific in the resistant LA3952.

Three growth and defense-associated hormones—zeatin (Figure 4A), indole-3-acetic acid (Figure 4C), and salicylic acid (Figure 4F), surprisingly remained constitutively low in the resistant LA3952 independent of the TPP-Lso infestation. Some hormones coexist in several isoforms with differential plant defense activity. For instance, zeatin, a cytokinin known to encourage lateral growth of buds [37]), has been shown to have an isomerically differential role in plant defense against microbial pathogens, where tobacco plants treated with exogenous trans-zeatin were significantly protected from damage by the pathogenic bacterium *Pseudomonas syringae*, while those treated with cis-zeatin were not [38]. We can speculate that such coexistence of isoforms in different concentrations may have led to the constitutively low abundances under certain treatments in the present study. In addition, although salicylic acid (SA) and jasmonic acid (JA) have been known to play important roles in the induced and systemic plant’s defense response to pathogenic attack and disease outbreak [39,40], sometimes, plants may deploy JA and ethylene in salicylic acid-independent endogenous signaling pathways in a manner that circumvents the production of, and signaling by, SA [41,42]. In fact, the seemingly antagonistic levels of SA and JA in the resistant LA3952 (Figure 4F,H) possibly might have a result of such crosstalk between the salicylic acid-dependent, and the salicylic acid-independent pathways.

Particularly puzzling was the dramatic detection of indole-3-acetic acid, IAA in the resistant LA3952 (Figure 4A), yet there was no observable breach in the subject test plants warrants further inquiry to determine if there might be a link with susceptibility and promotion of the pathogen growth associated with the overproduction of this hormone by some plants [43]. In tomato, knocking down IAA results in leaf and fruit deformation phenotypes [44]. However, we did not detect any odd phenotypes on LA3952 as expected on IAA deficient plants. Therefore, it is possible that the absence of detection may be due to technical limitations in the detection and quantification.

### 3.3. Volatile Metabolite Profiles Are Differentially Elicited on Susceptible and Resistant Plants in Response to TPP Vectoring or Not the Lso

Metabolomic approaches have been considered as efficient techniques to examine the profiles of metabolic changes in response to herbivore or pathogen infection, and to identify responsible metabolites associated with resistance or susceptibility [19,45,46,47]. Herbivore-induced plant volatiles have reportedly been involved in indirect defenses in responses to insect herbivory by acting as biochemical cues that attract natural predators [12]. These volatiles are mainly derived from terpenoids, fatty acid, and phenylalanine derivatives [11]. We identified 43 compounds in five main classes—fatty acid-derived, monoterpenes, norisoprenoids, phenylpropanoid-derived, and sesquiterpenes (Table 1 and Figure 5). Of the 43, ten chemical species showed some significant low or high levels in an either treatment- or genotype-specific manner. The FA-derived species, hexanal, and (E)-2-hexenal showed abundances of ~400% and 40%, respectively, higher in the resistant LA3952 plants treated with TPP-Lso(+) compared to the control and Lso(−) treatment. A norisoprenoid, β-Ionone was the only other compound to show a similar trend, albeit at a much-reduced abundance (Table 1). β-ionone promotes resistance of tobacco plants against pathogens by functioning as either a signaling molecule or an inducer of signal release involved in the plant defense mechanism [48,49]. Similarly, FA-derived green leaf volatiles (GLVs), such as hexanal and (E)-2-hexenal are reported to play protective roles in plant fitness by inhibiting the germination of plant pathogens [50,51,52]. Furthermore, pathogen-induced (E)-2-hexenal has been reported to possess antimycobacterial activity [53].

Strangely though, three monoterpenes—2-Carene, 3-Carene, and β-Phellandrene were each ~50% more abundant in the susceptible CM inoculated with Lso-free TPP, compared to either the control or the Lso(+) plants, even though these three compounds have variously been reported to participate in the defense against insect attack [54,55]. Still, such observations of the reversal of behavior may not be entirely new since many vector-borne bacterial pathogens are transmitted to the plant host by insect vectors, and this insect-bacteria-plant interaction may modulate both insect fitness and plant-host defensive signaling. The different metabolic profiles between susceptible and resistant plants and the defensive function may be attributed to the composition of terpenoids against phloem-feeding insects [24,56,57,58]. For instance, Lso could be implicated in modulating gene expression to alter the blend of volatiles, which may influence the vector behavior in order to increase the rate of inoculation and acquisition [15]. Moreover, the pathogen can interact with its vector directly and indirectly to take benefit of the host fitness [59].

This may have elicited distinct genetic and metabolic changes in plant hosts, which might correspond to a co-infestation status. For example, a study on tomato plants infected with TPP-Lso (−) had genes involved in plant defenses up-regulated regardless of the time-point, whereas TPP-Lso (+)-infected plants showed the initial down-regulation and delayed up-regulation of defense-related genes [9]. This is typical of many herbivore-induced plant volatiles (HIPVs), such as terpenoids [11]. In future experiments, timing will need to be staggered (beyond the 48 h in our experiment) in a manner that enables better determination of the eventual fate of actions by these monoterpenes.

It is equally strange that the abundances of phenylpropanoid (PA)-derived methylsalycylate, a herbivore-induced plant volatile (HIPV) well reported to favor entomopathogenicity in multi-trophic interactions [60], were significantly reduced in the LA_TPP-Lso(−) (2.78 ± 0.47) and in LA_TPP-Lso(−) (2.76 ± 0.22) compared to the CM_Control, CM_TPP-Lso(−), (CM_TPP-Lso(+), and the LA_Control (10.17 ± 1.52, 9.38 ± 1.08, 7.22 ± 1.14, and 5.99 ± 0.63, respectively). Three other PA-derivatives eugenol, benzophenone, and benzothiazole showed very similar trends though to a lesser extent, suggesting that these compounds may be susceptibility factors for TPP-Lso in the susceptible CM plants. In the present study, phenylpropanoids/benzenoids volatile metabolites were significantly decreased in plants infested with both TPP carrying the pathogen or not (Figure 5E). Others have previously observed suppression of plant defense reactions in pathogen-infected sugar beet (*Beta vulgaris*) leaves during the early disease development by the reduced gene expressions in the phenylpropanoids/benzenoid synthetic pathway [61]. Pathogens may promote the mutualism with their vectors by suppressing terpenoid synthesis [11,24]. This may elicit toxic or deterrent compounds to various types of organisms in the plant host, thereby improving the performance of vectors such that pathogens are better able to spread and blossom themselves onto plants. In addition, the metabolomics approach has been considered as a useful tool for screening and identifying biomarkers to understand metabolite alterations in response to the pathogen [62]. In this content, multivariate analysis using GC-MS dataset was performed to generate the variable importance for projection (VIP) scores (VIP > 1.0) derived from a PLS-DA model to screen the volatile metabolites, which were influenced by treatments (Figure S2).

Taken together, these findings imply that insect-susceptible and resistant tomato plants in response to Lso(−) or Lso(+) TPP may result in a different defensive reaction of HIVPs. Therefore, some of these metabolites can be possible pathogen-responsive biomarkers to distinguish susceptible and resistant tomato in response to TPP carrying Lso or not. However, these potential biomarkers need to be further investigated on more plant genotypes and at different growth stages.

## 4. Materials and Methods

### 4.1. Chemicals

Standards of plant hormones (abscisic acid (ABA), zeatin (ZA), gibberellic acid (GA), jasmonic acid (JA), salicylic acid (SA), and phenolic acids (4-hydroxy-benzoic acid, benzoic acid, caffeic acid, gallic acid, protocatechuic, and phthalic acid) were procured from Sigma (St. Louis, MO, USA). The plant hormone 12-oxo phytodienoic acid (OPDA) was obtained from Cayman Chemical (Ann Arbor, MI, USA). All other chemicals, including solvents of analytical and mass spec grade were obtained from Sigma-Aldrich (St. Louis, MO, USA).

### 4.2. Plant Materials and Experimental Design

The tomato TPP-susceptible cultivar CastleMart (CM) and the TPP-resistant recombinant inbred line LA3952 from the cross of *S. habrochaites* with *S. lycopersicum* [63], were grown at the Texas A&M AgriLife Research and Extension Center at Weslaco, TX, USA. Tomato plants were grown under controlled conditions with 16-h light/23 °C in 500-cc pots filled with peat moss Special Mix, BM Custom Blend (Berger Peat Moss Ltd., Saint-Modeste, QC, Canada). The full factorial experimental design consisted of two genotypes (CM vs. LA 3952) and three insect treatments [TPP-Lso(−), TPP-Lso(+), and a mock control]. The six factorial treatments were imposed at the 5th week after sowing. Ten adult psyllids were introduced onto the second fully opened leaf from the top, and confined on the leaf using organza bag cages to prevent them from escaping, while the mock-inoculated treatments consisted of empty cages only. Each treatment consisted of five replications. Local leaf tissues inside the bag cages were insects fed or mock-infected were collected 48 h after infestation, flash-frozen in liquid nitrogen, and stored at −80 °C until the time they were processed for analysis. The 48-h after infestation was chosen in accordance with previous studies on plant interactions with phloem feeders that have characterized resistance genotypes in tomato against potato aphid [64,65]. Furthermore, in the TPP-Lso complex, significant changes in transcript abundance has been detected 24 h post-inoculation [15]; therefore, changes in metabolic profile were expected to be observed 24–48 h period. Furthermore, previous studies using the electric penetration graph (EPG) indicate that at 24-h post-infection, 80% of the tested TPP had reached the inoculation access period (e.g., phloem salivation) [66]. The typical workflow is shown in Figure 1.

### 4.3. Insect Colonies

Lso-free TPP colonies and TPP colonies carrying Lso haplotype B of the Western biotype were reared in confining cages containing tomato and pepper plants, as previously reported [5]. The colonies were tested for Lso one day before infestation by PCR using the primer set OA2 forward 5′-GCGCTTATTTTTAATAGGAGCGGC-3′ [67], and OI2c reverse 5′-GCCTCGCGACTTCGCAACCCAT-3′ [68] targeting the 16S rRNA gene of Lso to detect its presence. Lso haplotype was also tested by PCR using SSR primer pairs Lso-SSR-1F forward 5′-TTATTTTGAGATGGTTTGTTAAATG-3′ and Lso-SSR-1R reverse 5′-TATTATCATTCTATTGCCTATTTCG-3′ [69]. The amplification was performed as described in the previous study [5].

### 4.4. Analysis of Plant Phenolics by UPLC/ESI-HR-QTOFMS

Frozen leaf materials were ground in liquid nitrogen, and 1 mL methanol was added to 50 mg of leaf sample. Each sample tube was vortexed (30 s), sonicated (1 h at 4 °C), and centrifuged (10,621× *g*) for 10 min. The supernatant was passed through 0.45 microfilters and injected into the UPLC/ESI-HR-QTOFMS, and the separation of phenolic acids was achieved using a published method [70]. The conditions for mass spectrometry and LC gradient separation were set following the methods by Kasote et al. [17]. The supernatant was injected into a UPLC/ESI-HR-QTOFMS equipped with Eclipse Plus C18 Rapid Resolution High Definition (1.8 μm, 50 × 2.1 mm) column. The gradient mobile phase, 0.1% aqueous formic acid (A) and 0.1% formic acid in acetonitrile (B) was used with the gradient program for pump B as follows—0–2 min, 0%; 2–15 min, 0–80%; 18–20 min, 80–100%. The separation was achieved at the flow rate of 0.2 mL min-1. Mass spectral analysis was performed in a high-resolution mass spectrometer (maXis impact, Bruker Daltonics, Bellerica, MA) using electrospray positive ionization mode. The operating parameters of the mass spectrometer were nebulizer gas pressure, 2.8 bar; nebulizer gas flow, 8 L min-1; sheath nebulizer gas temperature, 220 °C; sheath gas heater temperature, 220 °C. The data Analysis Software v4.3 was used to processes the data. The authentication standards of phenolic acids were used for quantitative profiling.

### 4.5. Estimation of Plant Phytohormones by UPLC/ESI-HR-QTOFMS

A set of 50 mg sample of freeze-dried and ground plant leaf tissues from each biological replicate was transferred into 1.5 mL microfuge tubes and 1 mL of 2-propanol: water: acetic acid (80:19:1, *v/v*) extraction solvent added to each tube. The samples were vortexed, sonicated (1 h), and centrifuged (10,621× *g*) for 10 min. The supernatant was separated, and the filtered samples were used for UPLC/ESI-HR-QTOFMS analysis using the authentication phytohormone standards. The separation of plant hormones was performed on the Eclipse Plus C_18_ RRHD column (1.8 μm, 50 × 2.1 mm) with a flow rate of 0.15 mL min^−1^ [17]. Authentic standard plant hormones were used to optimize the UPLC/ESI-HR-QTOFMS analysis conditions and to prepare calibration curves. The data Analysis Software v4.3 was used to quantify the data, according to our recent publications [62].

### 4.6. Analysis of Volatile Metabolic Profiles by HS-SPME/GC-MS

#### 4.6.1. Sample Preparation

Plant samples were ground in liquid nitrogen, and 100 mg were placed in 20 mL solid-phase microextraction (SPME) screw top amber vials with 1 mL of saturated calcium chloride, and 200 ng of camphor dissolved in ethanol as an internal standard [71]. The samples were vortexed for one min and sonicated for 30 min, before GC-MS analysis [72].

#### 4.6.2. HS-SPME/GC-MS Analysis Conditions

Tomato volatile compounds were extracted by headspace-solid phase microextraction (HS-SPME) equipped with a 50/30μm Carboxen/polydimethylsiloxane/divinylbenzene (CAR/PDMS/DVB) fiber (Sigma-Aldrich, St.Louis, MO) by modifying the conditions of our previous work [72]. The samples were incubated and extracted for 2 and 30 min at 60 °C, respectively. The SPME fiber was desorbed at 225 °C for 2 min, fiber conditioning was followed for 7 min, by placing it into the injector of gas chromatography equipped with an electron ionization source with a Dual-Stage Quadrupole (DSQ II) mass spectrometer (Thermo Scientific, Austin, TX, USA). Chromatographic separation was achieved with a Zebron ZB-5MS plus capillary column coated with 5% diphenyl-95% dimethylpolysiloxane (30 m × 0.25 mm) (Phenomenex, Inc. Torrance, CA, USA). The conditions applied for the GC-MS was an initial oven temperature of 40 °C, held for 1 min, then increased to 90 °C at a rate of 10 °C/min, and increased to 175 °C at a rate of 3 °C/min. Finally, it was increased to 230 °C at a rate of 35 °C/min, and held for 2 min at the final temperature, with a total run time of 38 min. The electron impact (EI) data from *m/z* 40 to 450 were acquired at a scanning speed of 11.5 scans per sec and with an ionization voltage of 70 eV. The ion source temperature and mass transfer line temperature were maintained at 280 °C. The data were recorded and processed using the Xcalibur software (v. 2.0.7., Thermo-Fisher Scientific, San Jose, CA, USA).

#### 4.6.3. Identification and Quantification Volatile Metabolites

Volatile organic compounds (VOCs) in samples were identified by comparing their mass spectra, Kovats retention indices (KI), and retention time of authentication standards. The KI values were determined using the number of carbons and their retention times of n-alkane standards (C_10_–C_24_), achieved from the same analysis condition as of samples. Each mass spectrum was also compared in Wiley 8 and NIST05 mass spectral library. The quantification of the relative changes in the volatile tomato metabolites was conducted using the internal standard, camphor, based on previous literature [18,73].

### 4.7. Statistical Analysis

In each treatment, five biological replications, each consisting of five technical replications, were analyzed using the coupling of liquid and gas chromatography to mass spectrum (LC-MS and GC-MS). The results were expressed as the mean of biological replicates ± standard error (SE). Statistical differences were evaluated between the technical replicates and between biological replicates before pooling the biological replicates for each tomato variety. The multiple means between the replicates, between varieties, and between treatments were compared using the Tukey’s Honest Significant Difference (HSD) after analysis of variance (ANOVA) with BM SPSS Statistics v. 23 (IBMCorp., Chicago, IL, USA). We also applied chemometric analysis on the LC-MS and GC-MS datasets using unsupervised principal components analysis (PCA), and supervised methods, such as partial least squares discriminant analysis (PLS-DA) and sparse partial least squares discriminant analysis (sPLS-DA) on the MetaboAnalyst 4.0. The PLS-DA method was used for discriminating the interaction effect of treatments on susceptible (CastleMart) and resistant (LA3952) tomato genotypes.

## 5. Conclusions

This study provides a detailed metabolite analysis in response to TPP and its vectored pathogen Lso in insect-susceptible and resistant tomato plants. We were able to identify metabolic changes in various classes of metabolites, including volatiles, hormones, and phenolics. Different volatile and non-volatile profiles were identified in susceptible and resistant genotypes in response to the tomato-potato psyllid vectoring or not, and its transmitted pathogen Lso. While some differentially regulated metabolites may be implicated in plant defensive signaling, further functional analysis (e.g., the use of hormone-deficient genotypes or exogenous applications) need to be performed to test the effect on insect mortality and fecundity. Moreover, upon validation, the identified metabolites could be used to screen and select breeding lines with enhanced resistance to TPP-Lso vector-pathogen complexes, which may reduce economic losses associated with the TPP insect and its transmitted disease.

## Figures and Tables

**Table 1:** Identification and quantification of volatile metabolites of mock, tomato-potato psyllid (TPP) without pathogen *Candidatus* Liberibacter solanacearum (Lso) (TPP-Lso (−)), and TPP carrying Lso (TPP-Lso (+)) infested insect-susceptible CatleMart (CM) and -resistant recombinant inbred line LA3952 (LA).

No.	RT	Compounds	Class of Volatile Metabolite	KI	ID	CM			LA		
						Control	TPP-Lso (−)	TPP-Lso (+)	Control	TPP-Lso (−)	TPP-Lso (+)
1	4.63	Hexanal	Fatty acid-derived	830	MS, KI, ST	10.57 ± 0.77a	3.96 ± 1.02b	1.48 ± 0.27b	2.57 ± 0.34b	2.72 ± 0.86b	14.92 ± 2.65a
2	5.71	(E)-2-Hexenal	Fatty acid-derived	872	MS, KI, ST	230.63 ± 22.94bc	223.99 ± 42.87bc	141.97 ± 17.01c	309.03 ± 43.91b	257.62 ± 31.03bc	492.11 ± 51.39a
3	7.5	2,4-Hexadienal	Fatty acid-derived	928	MS, KI	0.99 ± 0.19a	0.85 ± 0.17a	0.74 ± 0.16a	1.32 ± 0.38a	0.91 ± 0.17a	1.72 ± 0.57a
4	14.02	3,5-Octadien-2-one	Fatty acid-derived	1080	MS, KI	1.17 ± 0.24a	1.35 ± 0.25a	0.47 ± 0.10ab	1.23 ± 0.42a	0.21 ± 0.04b	0.73 ± 0.09ab
5	14.15	2-Octanol	Fatty acid-derived	1083	MS, KI	1.97 ± 0.25ab	2.61 ± 0.12a	1.27 ± 0.17bc	1.58 ± 0.41bc	0.75 ± 0.08c	1.00 ± 0.14c
6	20.81	Decanal	Fatty acid-derived	1197	MS, KI, ST	2.63 ± 0.14a	2.53 ± 0.10bc	2.41 ± 0.16bc	3.09 ± 0.27a	1.85 ± 0.11b	2.93 ± 0.22a
7	30.02	Dodecanal	Fatty acid-derived	1398	MS, KI	11.69 ± 0.96a	9.51 ± 0.66ab	9.30 ± 0.91ab	12.28 ± 1.06a	6.32 ± 0.44b	9.31 ± 0.55ab
8	38.21	Tetradecanal	Fatty acid-derived	1601	MS, KI	1.62 ± 0.12a	1.32 ± 0.12abc	1.18 ± 0.13bcd	1.60 ± 0.10ab	0.80 ± 0.05d	1.10 ± 0.07cd
9	8.13	α-Pinene	Monoterpene	944	MS, KI, ST	4.74 ± 0.44b	7.98 ± 1.12a	5.76 ± 0.92ab	4.42 ± 0.50b	6.40 ± 0.47ab	5.48 ± 0.62ab
10	10.19	β-Pinene	Monoterpene	986	MS, KI	6.45 ± 1.29c	9.96 ± 1.10bc	5.37 ± 1.06c	18.8 ± 1.31a	13.53 ± 2.01ab	14.60 ± 2.38ab
11	10.56	2-Carene	Monoterpene	996	MS, KI	93.43 ± 8.60a	141.57 ± 15.49a	103.56 ± 14.01a	103.45 ± 10.52a	103.99 ± 9.37a	114.66 ± 12.03a
12	10.86	3-Carene	Monoterpene	1004	MS, KI	40.80 ± 4.27a	62.07 ± 7.64a	47.89 ± 7.00a	47.42 ± 4.92a	44.15 ± 4.39a	45.18 ± 5.43a
13	11.41	α-Terpinene	Monoterpene	1019	MS, KI, ST	9.01 ± 1.30a	13.27 ± 1.69a	11.72 ± 1.75a	12.79 ± 0.39a	14.87 ± 1.58a	14.37 ± 1.55a
14	11.79	P-Cymene	Monoterpene	1029	MS, KI, ST	3.62 ± 0.58c	5.11 ± 0.68bc	3.50 ± 0.61c	4.25 ± 0.46c	10.58 ± 1.55a	8.55 ± 0.96ab
15	12.07	β-Phellandrene	Monoterpene	1036	MS, KI, ST	476.35 ± 40.23a	701.74 ± 75.28a	519.46 ± 74.65a	538.26 ± 51.54a	503.77 ± 39.98a	493.63 ± 47.56a
16	12.92	β-Ocimene	Monoterpene	1056	MS, KI	3.52 ± 0.33a	5.05 ± 0.53a	4.63 ± 0.99a	5.59 ± 0.83a	3.15 ± 0.41a	3.62 ± 0.47a
17	13.43	γ-Terpinen	Monoterpene	1067	MS, KI, ST	0.64 ± 0.08b	1.08 ± 0.11a	0.69 ± 0.10b	0.69 ± 0.09b	0.80 ± 0.05ab	0.74 ± 0.09ab
18	14.75	Terpinolen	Monoterpene	1095	MS, KI, ST	1.23 ± 0.15a	2.15 ± 0.30a	1.62 ± 0.33a	1.66 ± 0.20a	1.24 ± 0.14a	1.27 ± 0.15a
19	15.7	α-Campholenal	Monoterpene	1114	MS, KI	2.75 ± 0.09a	3.01 ± 0.14a	3.50 ± 0.62a	3.46 ± 0.15a	3.29 ± 0.11a	3.53 ± 0.05a
20	16.27	Alloocimene	Monoterpene	1124	MS, KI	0.43 ± 0.10abc	0.78 ± 0.15a	0.72 ± 0.12ab	0.39 ± 0.05abc	0.30 ± 0.04c	0.31 ± 0.05bc
21	22.32	Cuminal	Monoterpene	1234	MS, KI	0.67 ± 0.05b	1.01 ± 0.08a	1.14 ± 0.15a	0.66 ± 0.06b	0.17 ± 0.01c	0.31 ± 0.02c
22	22.85	Piperitone	Monoterpene	1247	MS, KI	0.43 ± 0.03a	0.42 ± 0.03ab	0.45 ± 0.06a	0.31 ± 0.03abc	0.28 ± 0.03bc	0.25 ± 0.03c
23	21.22	β-Cyclocitral	Norisoprenoids	1206	MS, KI, ST	4.53 ± 0.48bc	5.18 ± 0.74bc	3.17 ± 0.35c	6.33 ± 0.89b	4.29 ± 0.42bc	9.23 ± 0.94a
24	22.93	β-Cyclohomocitral	Norisoprenoids	1249	MS, KI	0.54 ± 0.07ab	0.60 ± 0.12ab	0.42 ± 0.02b	0.71 ± 0.10ab	0.42 ± 0.03b	0.79 ± 0.06a
25	30.39	α-Ionone	Norisoprenoids	1407	MS, KI	1.53 ± 0.22ab	1.75 ± 0.35ab	0.98 ± 0.08b	2.54 ± 0.34a	1.70 ± 0.23ab	2.56 ± 0.38a
26	32.71	β-Ionone	Norisoprenoids	1469	MS, KI, ST	18.77 ± 2.28abc	19.48 ± 2.70abc	11.33 ± 1.34c	22.01 ± 3.41ab	14.81 ± 1.42bc	26.93 ± 2.36a
27	32.83	β-Ionone-5,6-epoxide	Norisoprenoids	1472	MS, KI	2.37 ± 0.33bc	2.37 ± 0.43bc	1.53 ± 0.17c	3.26 ± 0.53ab	2.27 ± 0.23bc	4.11 ± 0.38a
28	44.68	Farnesylacetone	Norisoprenoids	1906	MS, KI, ST	0.25 ± 0.04a	0.16 ± 0.03a	0.37 ± 0.16a	0.20 ± 0.04a	0.17 ± 0.02a	0.19 ± 0.02a
29	19.9	Methyl salicylate	PA-derived	1184	MS, KI, ST	10.17 ± 1.52a	9.38 ± 1.08ab	7.22 ± 1.14ab	5.99 ± 0.63ab	2.78 ± 0.47b	2.76 ± 0.22b
30	21.34	Benzothiazole	PA-derived	1209	MS, KI, ST	2.97 ± 0.19a	2.90 ± 0.13a	2.66 ± 0.15a	2.91 ± 0.27a	1.36 ± 0.08b	1.35 ± 0.09b
31	23.52	Ethyl salicylate	PA-derived	1263	MS, KI	0.34 ± 0.08a	0.86 ± 0.40a	0.28 ± 0.03a	0.35 ± 0.07a	0.09 ± 0.03a	0.21 ± 0.05a
32	27.31	Eugenol	PA-derived	1345	MS, KI	2.09 ± 0.24a	1.59 ± 0.12ab	1.17 ± 0.18bcd	1.48 ± 0.23abc	0.64 ± 0.05d	0.85 ± 0.14cd
33	38.4	Benzophenone	PA-derived	1609	MS, KI	1.33 ± 0.07a	1.36 ± 0.03a	1.18 ± 0.07a	1.24 ± 0.07a	0.64 ± 0.04b	0.81 ± 0.04b
34	26.63	δ-Elemene	Sesquiterpenes	1331	MS, KI	2.91 ± 0.37cd	5.90 ± 0.81a	5.51 ± 0.79ab	3.30 ± 0.50bc	0.92 ± 0.42d	2.56 ± 0.26cd
35	29	β-Elemene	Sesquiterpenes	1379	MS, KI	3.07 ± 0.23bc	3.97 ± 0.37abc	2.83 ± 0.38c	4.49 ± 0.50a	3.02 ± 0.23bc	4.27 ± 0.25ab
36	30.17	β-Caryophyllene	Sesquiterpenes	1401	MS, KI, ST	29.97 ± 4.09b	48.28 ± 5.07ab	42.93 ± 7.83ab	56.07 ± 7.73a	36.42 ± 4.14ab	37.16 ± 2.95ab
37	30.71	γ-Elemene	Sesquiterpenes	1416	MS, KI	1.10 ± 0.16ab	1.37 ± 0.24ab	0.75 ± 0.10b	1.71 ± 0.34b	1.22 ± 0.19ab	1.19 ± 0.25ab
38	31.15	Aristolene	Sesquiterpenes	1428	MS, KI	1.10 ± 0.15a	1.10 ± 0.14a	0.97 ± 0.17ab	0.98 ± 0.12ab	0.55 ± 0.06b	0.57 ± 0.05b
39	31.67	α-Humulene	Sesquiterpenes	1442	MS, KI	8.54 ± 1.24b	11.76 ± 1.24ab	11.99 ± 2.11ab	14.59 ± 1.61a	9.49 ± 1.04ab	9.44 ± 0.77ab
40	33.04	Valencene	Sesquiterpenes	1478	MS, KI, ST	0.35 ± 0.038a	0.50 ± 0.07a	0.51 ± 0.11a	0.35 ± 0.05a	0.26 ± 0.03a	0.28 ± 0.03a
41	33.74	β-Guaiene	Sesquiterpenes	1495	MS, KI	0.70 ± 0.06ab	0.93 ± 0.09a	0.91 ± 0.18a	0.55 ± 0.10ab	0.33 ± 0.08b	0.45 ± 0.07b
42	34.41	Dihydroactinidiolide	Sesquiterpenes	1512	MS, KI	2.46 ± 0.37a	2.27 ± 0.37a	1.80 ± 0.22a	2.50 ± 0.42a	2.08 ± 0.22a	3.17 ± 0.43a
43	36.68	Caryophyllene Oxide	Sesquiterpenes	1566	MS, KI	0.72 ± 0.12c	0.99 ± 0.13c	0.70 ± 0.12c	1.83 ± 0.25a	1.18 ± 0.12bc	1.63 ± 0.17ab

KI: Retention index, relative to n-alkanes (C_8_–C_24_) on the ZB-5 capillary column; ID: Identification methods, MS: Mass spectra; KI values that agreed with the data reported in previous literature or the database on the web (http://www.nist.gov); ST: Standard comparison, compounds identified using authentic standards. PA: phenylpropanoid; Different letters in the same row indicated significant differences between treatment at 95%. Results were expressed as mean ± S.E. (ng/g of fresh weight).

## Supplementary Materials

The following are available online at https://www.mdpi.com/2223-7747/9/9/1154/s1, Figure S1: The concentration of volatile metabolites according to the impact of the genotype and treatment, and the asterisk indicates the significant difference based on the student t-test and posthoc Tukey’s test (*, *p* < 0.05; **, *p* < 0.01; and ***, *p* < 0.001). n.s. indicates no significance. Figure S2: Multivariate analysis using a gas chromatography–mass spectrometry (GC-MS) dataset to explore the different effects of the mock control, tomato-potato psyllid (TPP) without *Candidatus* Liberibacter solanacearum (Lso) (TPP-Lso (−)), and TPP carrying Lso (TPP-Lso (+)) inoculations on the insect-susceptible and -resistant tomato varieties, CastleMart (CM) and recombinant inbred lines LA3952 (LA), respectively. Score plots of (A) principal component analysis (PCA), (B) partial least squares discriminant analysis (PLS-DA), and (C) sparse PLS-DA indicate the discrimination between studied test groups. (D) Variable importance for projection (VIP) scores derived from a PLS-DA model to examine and filter the variables (VIP > 1.0) having influence on the PLS-DA scores plot. (E) The loading plot illustrates the variables responsible for the separating pattern in the PLS-DA model. Table S1: Identification of plant phenolics and serotonin in tomatoes by an ultra-performance liquid chromatography coupled to electrospray ionization high resolution quadrupole time-of flight mass spectrometry (UPLC/ESI-HR-QTOFMS) with diode array detector (DAD) in ESI positive ionization mode. Table S2: Identification of plant hormones and melatonin using an ultra-performance liquid chromatography coupled to electrospray ionization high resolution quadrupole time-of flight mass spectrometry (UPLC/ESI-HR-QTOFMS).
