# Supplementary material for: Tomato Metabolic Changes in Response to Tomato-Potato Psyllid (Bactericera cockerelli) and Its Vectored Pathogen Candidatus Liberibacter solanacearum
Source: Plants (Basel). 2020 Sep 6;9(9):1154. doi: 10.3390/plants9091154 (PMC7570104; doi:10.3390/plants9091154)
Supplement: Supplementary file 1 [file plants-09-01154-s001.pdf]

Supporting Information

Tomato metabolic changes in response to tomato-potato psyllid (*Bactericera cockerelli*) and its vectored pathogen *Candidatus* Liberibacter solanacearum

Jisun H.J. Lee[1,2‡], Henry O. Awika[3‡], Guddadarangavvanahally K. Jayaprakasha[1,2], Carlos A. Avila[2,3*], Kevin M. Crosby[1,2*], and Bhimanagouda S. Patil[1,2*]

1 Vegetable and Fruit Improvement Center, Texas A&M University, 1500 Research Parkway, A120, College Station, TX 77845-2119, USA; jslee@tamu.edu (J.H.J.L), gkjp@tamu.edu (G.k.J),

2 Department of Horticultural Sciences, Texas A&M University, College Station, TX 77843, USA

3 Texas A&M AgriLife Research and Extension Center, 2415 E Hwy 83, Weslaco, TX 78596, USA; Henry.Awika@ag.tamu.edu (H.O.A)

* Correspondence: b-patil@tamu.edu (B.S.P), k-crosby@tamu.edu (K.M.C), and Carlos.Avila@ag.tamu.edu (C.A.A)

‡ Author contributed equally to this work.

Table S1. Identification of plant phenolics and serotonin in tomatoes by an ultra-performance liquid chromatography coupled to electrospray ionization high resolution quadrupole time-of flight mass spectrometry (UPLC/ESI-HR-QTOFMS) with diode array detector (DAD) in ESI positive ionization mode.

**Rt (min)**	**Compound**	**Experimental mass (*m/z*)**	**UV λmax (nm)**	**MS/MS fragments at positive mode (*m/z*)**	**Molecular formula**	**Experimental mass (*m/z*)**	**Theoretical mass (m/z)**	**Mass error (ppm)**
1.1	Gallic acid	171.03	270	153, 107	$C_7H_6O_5$	171.03	171.03	6.4
1.4	Serotonin	177.10	274	160, 117, 115	$C_{10}H_{12}N_2O$	177.10	177.10	6.0
2.1	Protocatechuic acid	155.03	258	137, 84	$C_7H_6O_4$	155.03	155.03	4.0
3.5	4-hydroxybenzoic acid	139.04	254	121, 84	$C_7H_6O_3$	139.04	139.04	5.0
5.3	Phtalic acid	167.03	279	149	$C_8H_6O_4$	167.03	167.03	-6.0
6.4	Chlorogenic acid	355.10	326	163, 135, 117, 89	$C_{16}H_{18}O_9$	355.10	355.10	6.5
7.4	p-Coumaric acid	165.06	308	119, 91	$C_9H_8O_3$	165.06	165.05	6.7
9.2	Ferulic acid	195.07	322	177, 117, 89	$C_{10}H_{10}O_4$	195.07	195.07	3.6
10.9	Rutin	611.16	354	303	$C_{27}H_{30}O_{16}$	611.16	611.16	6.9
15.9	Naringenin	273.08	289	153	$C_{15}H_{12}O_5$	273.08	273.08	8.8

Table S2. Identification of plant hormones and melatonin using an ultra-performance liquid chromatography coupled to electrospray ionization high resolution quadrupole time-of flight mass spectrometry (UPLC/ESI-HR-QTOFMS).

Rt (min)	Compound	Molecular formula	Theoretical mass (m/z)	Experimental mass (m/z)	Mass error (ppm)
4.3	Zeatin	$C_{10}H_{13}N_5O$	220.12	220.12	0.0
6.4	Gibberellin	$C_{19}H_{22}O_6$	347.15	347.15	-2.9
7.2	Melatonin	$C_{13}H_{16}N_2O_2$	233.13	233.13	-0.9
7.6	Indole-3-acetic acid	$C_{10}H_9NO_2$	176.07	176.07	-4.5
7.8	Salicylic acid	$C_7H_6O_3$	139.04	139.04	-5.8
7.8	Abscisic acid	$C_{15}H_{20}O_4$	265.14	265.14	-1.5
8.8	Jasmonic Acid	$C_{12}H_{18}O_3$	211.13	211.13	-1.4
11.9	12-Oxo-phytodienoic acid	$C_{18}H_{28}O_3$	293.21	293.21	-0.3

Fig. S1

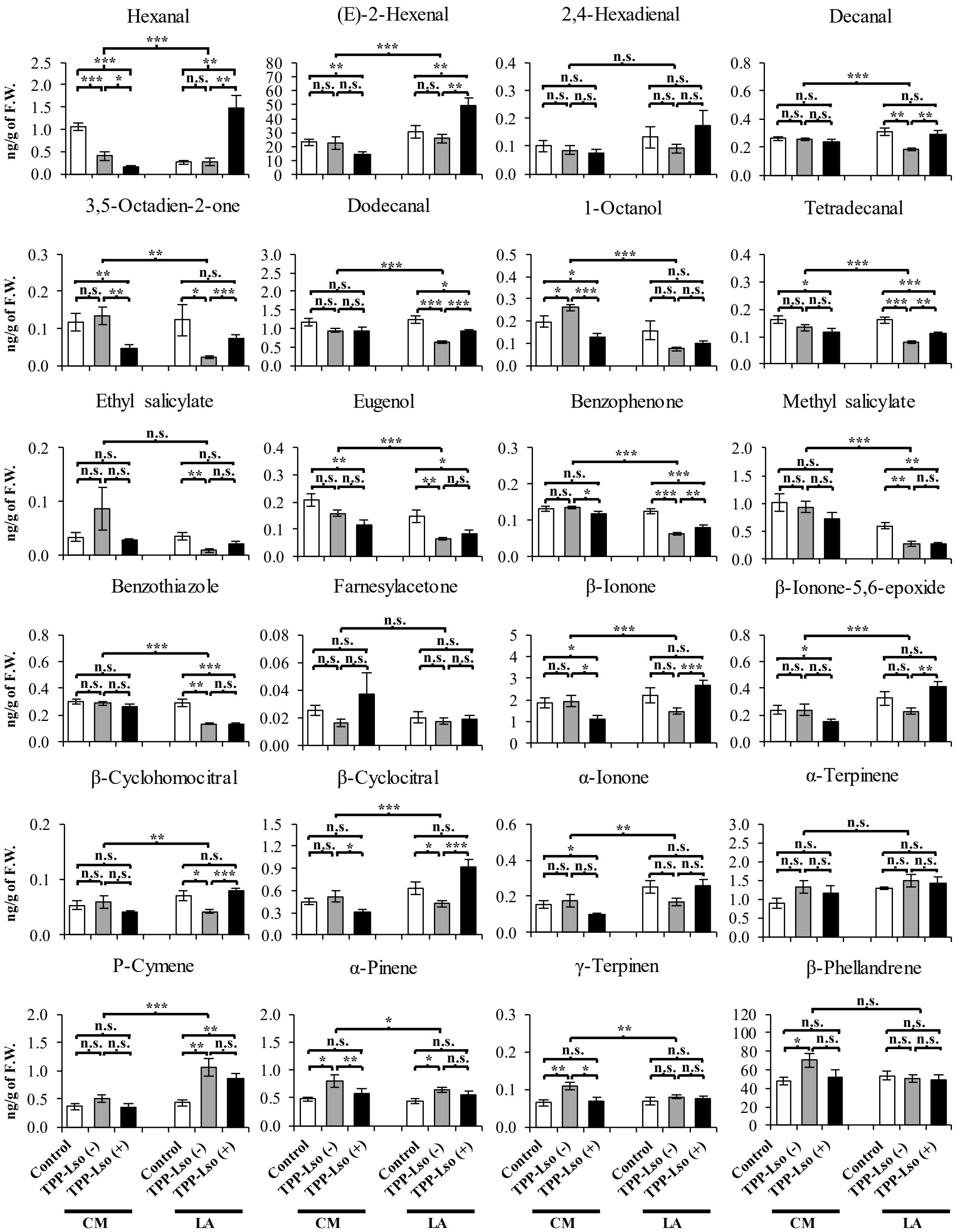
Hexanal
(E)-2-Hexenal
2,4-Hexadienal
Decanal
3,5-Octadien-2-one
Dodecanal
1-Octanol
Tetradecanal
Ethyl salicylate
Eugenol
Benzophenone
Methyl salicylate
Benzothiazole
Farnesylacetone
β-Ionone
β-Ionone-5,6-epoxide
β-Cyclohomocitral
β-Cyclocitral
α-Ionone
α-Terpinene
P-Cymene
α-Pinene
γ-Terpinen
β-Phellandrene
ng/g of F.W.
Control
TPP-Lso (-)
TPP-Lso (+)
CM
LA

(continued)

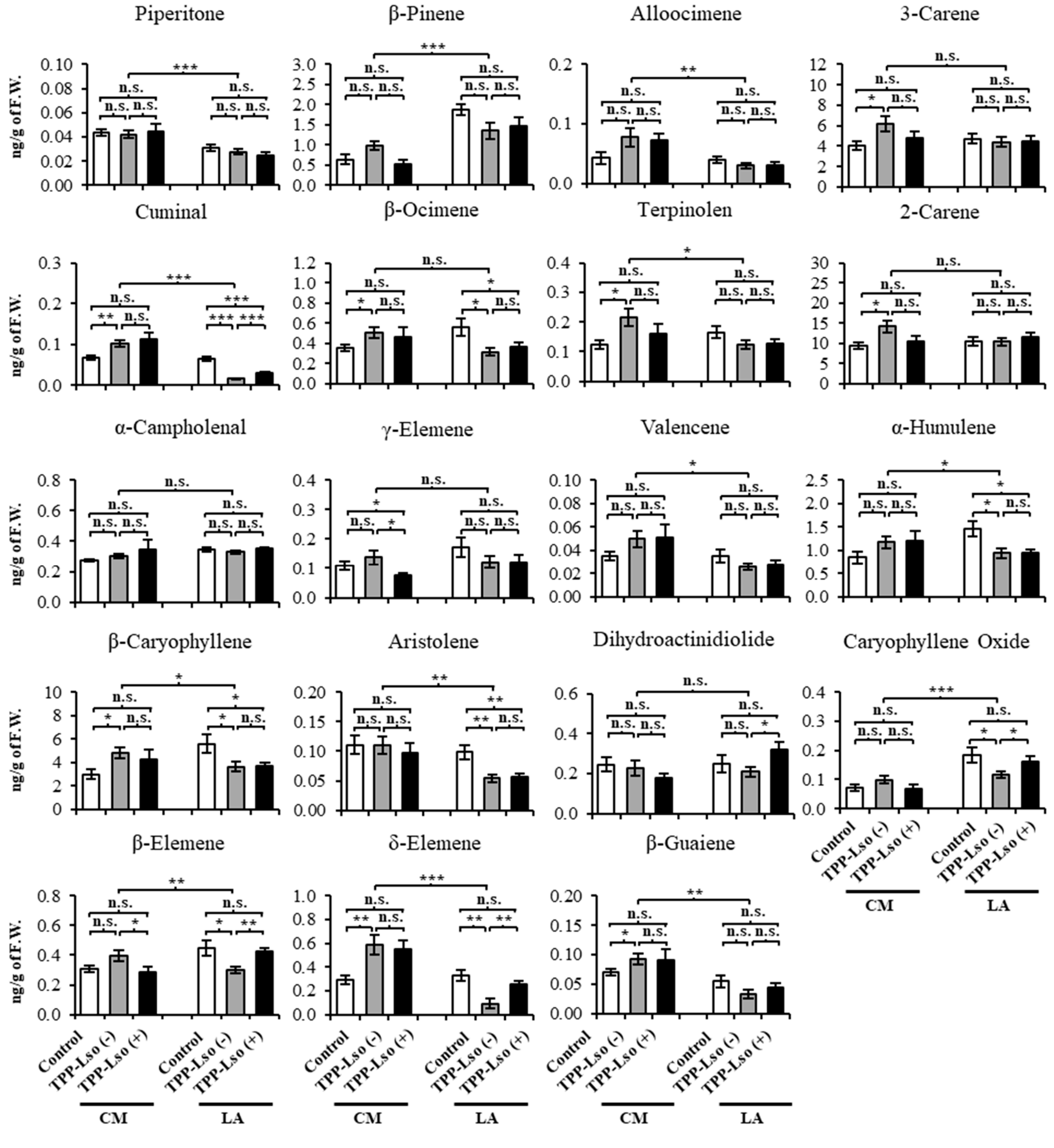

Figure S1. The concentration of volatile metabolites according to the impact of the genotype and treatment, and the asterisk indicates the significant difference based on the student t-test and posthoc Tukey's test (*, P < 0.05; **, P < 0.01; and ***, P < 0.001). n.s. indicates no significance.

Fig. S2

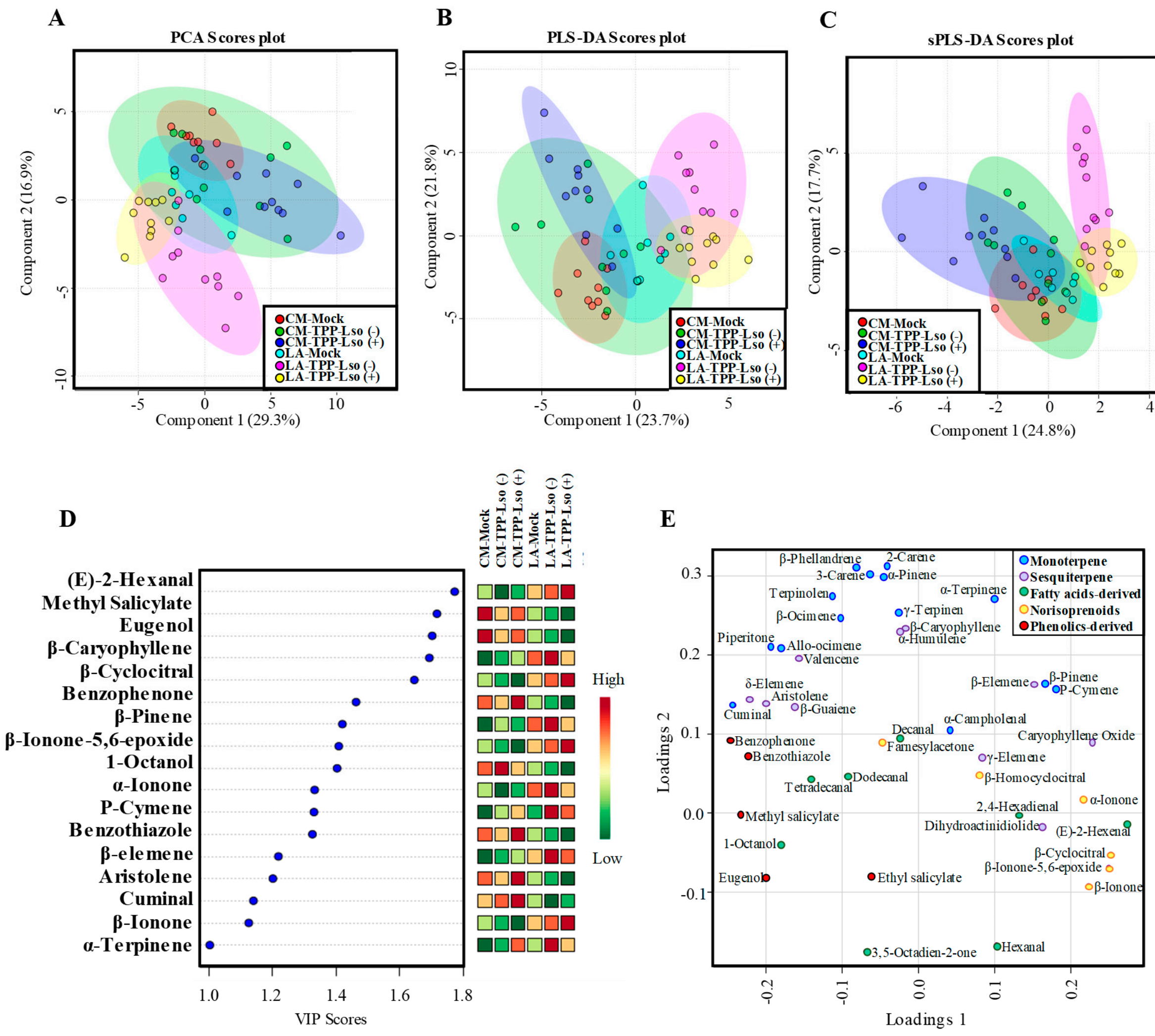

Figure S2. Multivariate analysis using a gas chromatography–mass spectrometry (GC-MS) dataset to explore the different effects of the mock control, tomato-potato psyllid (TPP) without *Candidatus* Liberibacter solanacearum (Lso) (TPP-Lso (-)), and TPP carrying Lso (TPP-Lso (+)) inoculations on the insect-susceptible and -resistant tomato varieties, CastleMart (CM) and recombinant inbred lines LA3952 (LA), respectively. Score plots of (A) principal component analysis (PCA), (B) partial least squares discriminant analysis (PLS-DA), and (C) sparse PLS-DA indicate the discrimination between studied test groups. (D) Variable importance for projection (VIP) scores derived from a PLS-DA model to examine and filter the variables (VIP > 1.0) having influence on the PLS-DA scores plot. (E) The loading plot illustrates the variables responsible for the separating pattern in the PLS-DA model.
